# Interaction between NKG2D and its ligands MICA/B activates the DAP12/SYK/p53/p21 axis to drive pulmonary fibrosis

**DOI:** 10.3389/fimmu.2026.1770733

**Published:** 2026-03-02

**Authors:** Caiping Zhao, Hong Ren, Qingming Ke, Qiuzhu Chen, Jinlian He, Ruimin Tian, Hudan Pan, Liang Liu

**Affiliations:** 1 State Key Laboratory of Traditional Chinese Medicine Syndrome, The Second Affiliated Hospital of Guangzhou University of Chinese Medicine, Guangzhou, China; 2 Shenzhen Hospital of Integrated Traditional Chinese and Western Medicine, Shenzhen, China; 3 Chinese Medicine Guangdong Laboratory (Hengqin Laboratory), Guangdong-Macao In-Depth Cooperation Zone in Hengqin, Zhuhai, China; 4 Guangzhou National Laboratory, Guangzhou, China

**Keywords:** cellular senescence, MICA/B, NKG2D, pulmonary fibrosis, SYK

## Abstract

**Background:**

Pulmonary fibrosis (PF) is a progressive, fatal interstitial lung disease with limited therapeutic options. Emerging evidence implicates immune–fibrotic crosstalk in PF pathogenesis, although the underlying molecular mechanisms remain poorly defined. While Natural Killer (NK) cells and their activating receptor NKG2D have been linked to fibrotic processes, their functional role in PF is unclear. This study investigates the NKG2D-DAP12-SYK-p53-p21 signaling axis as a potential driver of PF through immune-fibroblast interactions.

**Methods:**

We characterized the dynamic expression profile of NKG2D in pulmonary tissues derived from bleomycin (BLM)-induced model mice. Mechanistic investigations utilized AAV5-mediated NKG2D overexpression systems, coimmunoprecipitation assays, and functional pathway dissection to elucidate the DAP12-SYK-p53-p21 signaling axis. Therapeutic efficacy was evaluated via anti-NKG2D antibody treatment in murine PF models via histopathology, micro-CT imaging, and molecular profiling of fibrosis markers (collagen-I, fibronectin) and senescence-associated proteins (p-p53, p21).

**Results:**

Significant upregulation of NKG2D on pulmonary NK cells and its ligands on fibroblasts was detected in murine PF. AAV5-mediated NKG2D overexpression exacerbated BLM induced fibrosis, as evidenced by increased fibrosis scores alongside elevated levels of collagen-I and fibronectin. Mechanistically, NKG2D activation triggered DAP12-dependent SYK activation, leading to p53 phosphorylation and p21-mediated cellular senescence. Treatment with anti-NKG2D antibodies effectively mitigated disease progression by reducing collagen deposition while suppressing the downstream expression of SYK and p21.

**Conclusion:**

This study proposes that the NKG2D-DAP12-SYK-p53-p21 axis may represent a novel pathogenic pathway in PF, potentially linking immune dysregulation to cellular senescence. Therapeutic targeting of NKG2D could thus hold promise for the concurrent modulation of immune-fibrotic crosstalk and fibrotic progression, which might offer a new strategic direction for PF management.

## 1 Introduction

Pulmonary fibrosis (PF) is a progressive and fatal interstitial lung disease characterized by irreversible scarring of lung tissue, leading to respiratory failure and high mortality (1). A registry-based study conducted in the United States reported an annual incidence of 13.0 cases per 100 patient-years with PF (2). PF has a median survival of 2.5–3.5 years post-diagnosis, with a 5-year survival rate of less than 30% (3), which is worse than that of many other cancers. With the aging population, the incidence of PF continues to rise, yet its exact pathogenesis remains unclear, and treatment options remain limited. Current therapeutic paradigms rely on antifibrotic agents such as nintedanib and pirfenidone, which modestly slow disease progression but fail to reverse established fibrosis (4). However, these agents impose a substantial economic burden and carry systemic side effects that constrain their clinical applicability (4, 5). Moreover, traditional immunosuppressive therapies (e.g., corticosteroids) have failed to improve outcomes and may even worsen prognosis, suggesting that PF is not merely an inflammatory disorder but rather a maladaptive repair process driven by aberrant immune–fibrotic crosstalk (6, 7). Therefore, targeting key immune cell subpopulations in the immune microenvironment may become a paradigm shift therapeutic strategy.

Natural killer (NK) cells are increasingly recognized as key regulators of fibrotic responses through their cytotoxic and cytokine-secreting functions (8, 9). NKG2D is a major activating receptor on NK cells that recognizes stress-induced ligands, such as MICA/B and ULBP family proteins (10, 11). This study focuses on the NKG2D ligands MICA and MICB (MICA/B). Its signal is transduced through the adaptor proteins DAP10 and DAP12, leading to the activation of downstream pathways, including spleen tyrosine kinase (SYK), which regulates cytotoxicity and cytokine production. Under chronic inflammatory conditions, persistent NKG2D activation may exacerbate tissue damage (11, 12). Notably, recent evidence suggests that NKG2D and its signaling pathway may be involved in regulating cellular senescence, a fundamental biological process critical in aging and age-related diseases, including fibrosis (13, 14). Senescent cells accumulate in fibrotic tissues and drive fibroblast activation and extracellular matrix deposition via paracrine signaling, such as the senescence-associated secretory phenotype (SASP) (15). NKG2D ligands are upregulated on senescent cells, and NK cell-mediated clearance of these cells partially depends on NKG2D signaling (16). Of particular note, SYK has been demonstrated to phosphorylate p53 and upregulate the cyclin-dependent kinase inhibitor p21, thereby inducing p53-mediated cellular senescence (17–19). This suggests that in pulmonary fibrosis, the NKG2D-DAP12-SYK signaling axis may drive cellular senescence by activating the p53/p21 pathway.

Currently, our understanding of the mechanism by which NKG2D regulates cellular senescence in the context of PF remains limited. This study aims to address this knowledge gap by investigating whether the NKG2D-MICA/B axis drives disease progression through the aforementioned signaling pathway. We hypothesize that the NKG2D-DAP12-SYK-p53 signaling pathway drives cellular senescence in PF, thereby positioning NKG2D as a novel immune-fibrotic checkpoint. The findings from this study will provide a novel therapeutic target for PF and offer preclinical rationale for anti-NKG2D monoclonal antibodies as a promising immunotherapeutic strategy.

## 2 Methods

### 2.1 Construction of a PF mouse model and detection of NKG2D-MICA/B expression

To investigate the role of the NKG2D-MICA/B axis in pulmonary fibrosis, we employed a well-established bleomycin (BLM)-induced mouse model. Male C57BL/6 mice (8–10 weeks old, 20–25 g, n=20) were anesthetized with pentobarbital sodium and subsequently administered BLM (1.5 mg/kg in 40 μL of PBS, n=10) or PBS via intratracheal instillation (n=10). Lung tissues were harvested 21 days post-induction for NKG2D-MICA/B detection via Western blot, RT–qPCR, and flow cytometry. To confirm the successful construction of the pulmonary fibrosis model, we examined the expression of fibronectin protein and hydroxyproline in mouse lung tissue. For flow cytometry analysis, NKG2D^+^ cells were gated as CD3^-^NK1.1^+^NKG2D^+^ and subjected to semi-quantitative counting.

### 2.2 Cellular interaction between pulmonary fibroblasts and NK cells

To study the cellular crosstalk critical to PF pathogenesis, we utilized an *in vitro* coculture system. Human NK-92MI cells were activated with IL-2 (100 IU/mL) and co-cultured with mitomycin C-treated K562 cells, a standard protocol to enhance NK cell effector functions, prior to coculture with fibroblasts. To explore the underlying mechanism, we first performed *in vitro* functional validation using the human lung fibroblast cell line MRC-5. MRC-5 were transfected with a MICA-overexpressing plasmid (PLVX-IRES-ZsGreen1-MICA-Stbl3, 2 μg/mL) via Lipofectamine 3000 (Invitrogen). Transfected MRC-5 cells were cocultured with NK-92MI cells (1:5 ratio) in RPMI-1640 medium supplemented with 10% FBS for 48 hours. NKG2D expression on NK-92MI cells was analyzed by flow cytometry.

To validate our findings in a model with greater physiological relevance to human lung fibrosis, we extended our studies to human fetal lung fibroblasts (HFL-1 cells). HFL-1 cells were pretreated with recombinant human TGF-β1 (5 ng/mL) for 24 hours to induce a profibrotic phenotype, simulating a key aspect of the fibrotic microenvironment. These activated fibroblasts were subsequently cocultured with pre-activated NK-92MI cells at a 1:5 ratio in serum-free DMEM for 72 hours. Following coculture, fibroblast expression of the NKG2D ligand MICB and classic fibrosis markers (including fibronectin and TGF-β1) was analyzed by Western blot and/or RT–qPCR. Concurrently, NK cell effector functions were evaluated by measuring the secretion of interferon-gamma (IFN-γ) by ELISA and cytolytic activity via a lactate dehydrogenase (LDH) release assay.

### 2.3 Generation of NKG2D-AAV5 mice and detection of fibrosis-related indicators

Adeno-associated virus 5 (AAV5) exhibits optimal pulmonary tropism among serotypes (AAV2/5/6/9) because of enhanced lung infection efficiency. AAV5-mediated NKG2D overexpression was achieved via a first-generation adenovirus system. E1/E3-deleted Ad5 plasmids were linearized with PacI, transfected into HEK293 cells (E1-expressing), and purified via CsCl gradient centrifugation. We obtained the following two adeno-associated viruses: pAAV[Exp] (20)-CAG>mKlrk1[NM_033078.4](ns):T2A:EGFP: WPRE, pAAV[Exp]-CAG>EGFP: WPRE. Male C57BL/6 mice (6-8 weeks old) were randomly assigned to six groups: the control group, BLM group, AAV5-EGFP group, AAV5-EGFP+BLM group, NKG2D-AAV5 group and NKG2D-AAV5+BLM group, with 10 mice in each group. On day 0, the mice were intratracheally administered 50 μL of empty vector or AAV5-NKG2D (2*10^11^vg/ml). On day 14, BLM (0.75 mg/kg, **Supplementary Figure 1**.) or saline was delivered via intratracheal injection. Daily body weight monitoring was conducted. On day 35 (3 weeks post-BLM), the mice underwent pre-sampling CT imaging.

To evaluate the safety profile of AAV5, liver, spleen, and kidney tissues were harvested and weighed. For detailed characterization of the transduction efficiency and targeting specificity of AAV5, 3-μm-thick lung tissue sections were subjected to immunofluorescence (IF) staining to detect NKG2D expression, thereby directly validating the efficacy of AAV5-mediated gene delivery. Subsequently, histopathological assessments were performed on lung sections using hematoxylin and eosin (HE) staining and Masson’s trichrome staining to evaluate the severity of pulmonary fibrosis and inflammatory infiltration. Furthermore, total protein was extracted from the right lung tissue for the detection of fibrosis-related markers. Western blot (WB) analysis was conducted to examine the expression levels of the NKG2D receptor protein, as well as fibrosis indicators including collagen type I and fibronectin. These results provide additional protein-level confirmation of AAV5 transduction and its regulatory effects on the fibrotic process.

### 2.4 NKG2D-AAV5 induced cellular aging in mice

To elucidate the mechanism of action of NKG2D-AAV5 and assess the expression of downstream signaling molecules in the BLM-induced pulmonary fibrosis model, the following experiments were performed: DAP12 protein expression in lung tissue was determined. The interaction between NKG2D and DAP12 was analyzed via immunofluorescence colocalization and coimmunoprecipitation (co-IP). In addition, downstream signaling involves SYK detection (IHC) and aging-related proteins (p-p53, p21) via WB.

### 2.5 NKG2D antibody intervention in PF mice validates the role of NKG2D in PF

To validate the role of NKG2D in pulmonary fibrosis, we treated PF mice with NKG2D antibodies. Male C57BL/6 mice (8–10 weeks old) were subjected to a BLM-induced PF model via single intratracheal instillation of BLM (1.5 mg/kg). On day 0, the mice were randomized into four groups: the control group (n=10), model group (n=12), PFD group (300 mg/kg, qd, n=12), and NKG2D-Ab group (100 μg (21, 22), qod, n=12). Drug interventions were administered accordingly starting from day 1 and continued consecutively for 21 days. Lung tissues and bronchoalveolar lavage fluid (BALF) were collected on day 21. Fibrosis severity was assessed via Masson’s trichrome staining, high-resolution micro-CT imaging, and collagen deposition analysis via western blotting (Fibronectin). Concurrently, inflammation was evaluated through BALF cell counts and histopathological examination via H&E staining. Furthermore, the protein expression of DAP12, SYK, and p21 in lung tissues was determined by IF, IHC, and WB. To clarify the regulatory role of SYK on p53, this study treated HEK293T cells overexpressing p53 with the SYK inhibitor R406 (10 μM), and then detected changes in p53 protein levels by WB.

### 2.6 Statistics

Statistical analyses were performed using GraphPad Prism (version 9.0; San Diego, CA, USA) and R (version 4.3.1). Data are presented as the mean ± standard error of the mean (SEM). For comparisons between two groups, an unpaired two-tailed Student’s t−test was used after confirming normality with the Shapiro–Wilk test and homogeneity of variances with F−test; otherwise, the non−parametric Mann–Whitney U test was applied. For comparisons among multiple groups, one−way analysis of variance (ANOVA) was used when data met assumptions of normality and equal variance. If the ANOVA showed a significant overall effect (P < 0.05), post−hoc comparisons were conducted using Tukey’s honestly significant difference (HSD) test for all pairwise comparisons, or Dunnett’s test when comparing each treatment group against a single control group. A P−value < 0.05 was considered statistically significant.

## 3 Results

### 3.1 Upregulation of NKG2D on pulmonary NK cells characterizes fibrotic lung pathology

**Figure 1:**
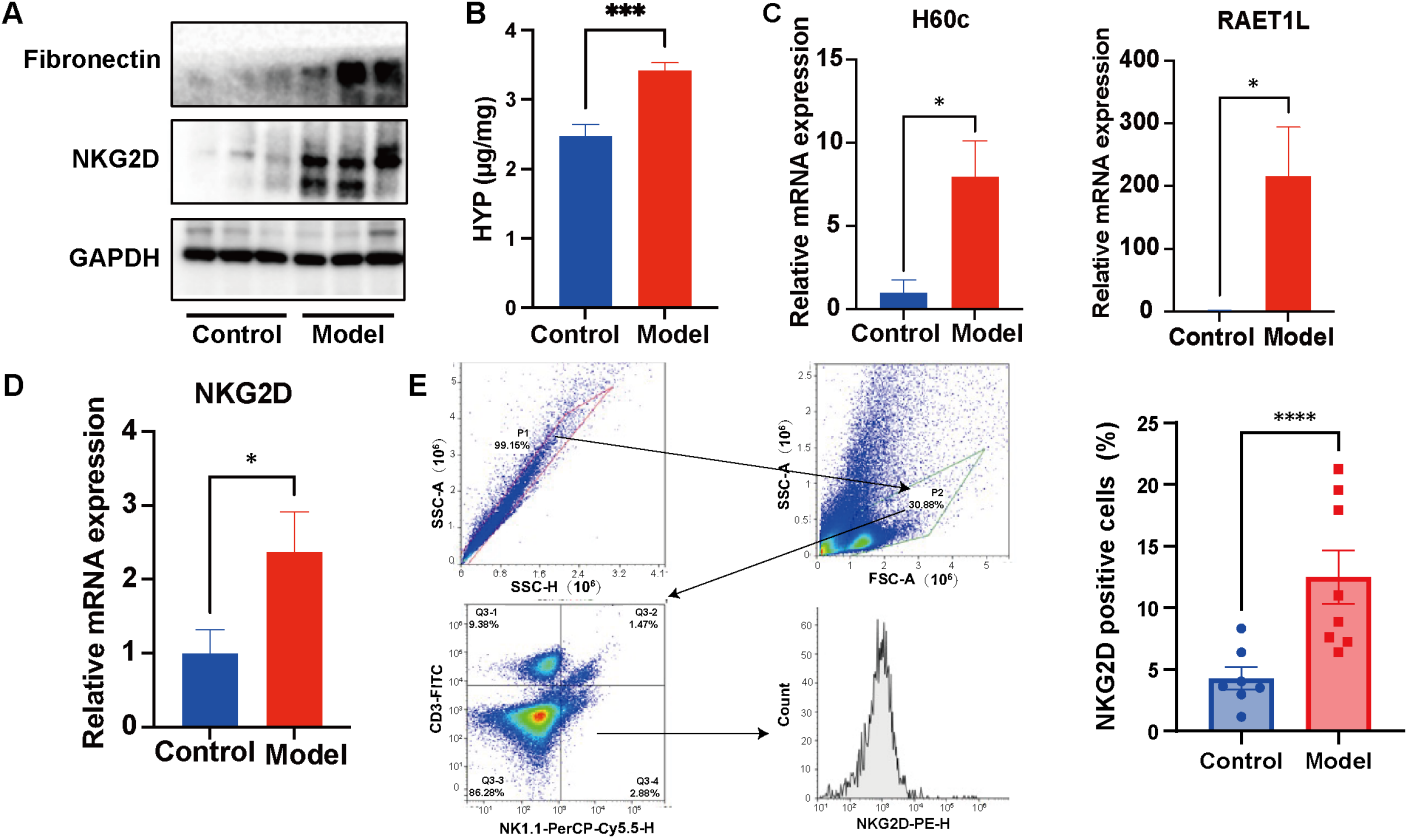
Upregulation of NKG2D on pulmonary NK cells characterizes fibrotic lung pathology. **(A, B)** The expression of fibronectin and the content of hydroxyproline in the lung tissue of the pulmonary fibrosis mice.(n=3/8) **(A, C, D)** The protein and mRNA expression of NKG2D and its ligands in BLM-induced murine PF lungs. (n=3) **(E)** The percentage of NKG2D-positive cells in the lung tissue of BLM-induced PF mice was detected by flow cytometry. (n=7/8) In mice, the NKG2D ligands H60c and RAET1L are functional homologs of the human MICA/B proteins studied here. The data are presented as the mean ± SEMs; *P<0.05, **P<0.01, ***P<0.001 vs. Model group.

Compared with the control group, the expression of fibronectin in the lung tissue of the model group was upregulated, and the content of hydroxyproline, a collagen-characteristic amino acid, was significantly increased, indicating that the animal model of pulmonary fibrosis was successfully constructed (**Figures 1A, B**). Both mRNA and protein levels of NKG2D and its ligands were significantly elevated in lung tissues compared to controls (**Figures 1A, C, D**). In mice, the NKG2D ligands H60c and RAET1L are functional homologs of the human MICA/B proteins studied here. Flow cytometric analysis confirmed a pronounced accumulation of NKG2D^+^ NK cells in fibrotic lungs (**Figure 1E**). Together, these data demonstrate a dysregulated NKG2D-MICA/B axis during PF pathogenesis.

### 3.2 NKG2D-mediated crosstalk potentiates profibrotic NK-fibroblast interactions

**Figure 2:**
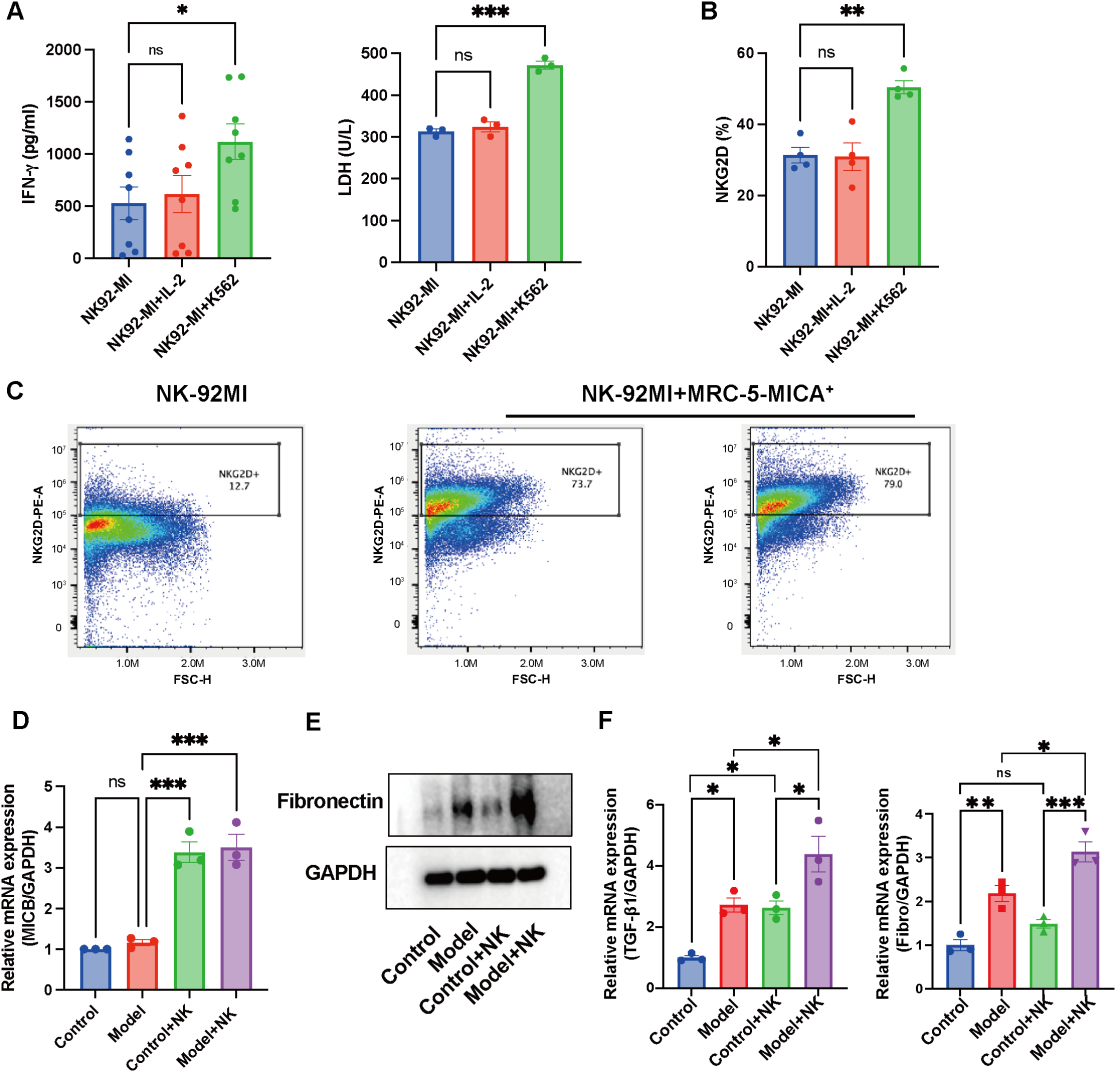
NKG2D-mediated crosstalk potentiates pro-fibrotic NK-fibroblast interactions. **(A)** Detection of IFN-γ and LDH expression in NK-92MI cells following IL-2 stimulation or co-culture with K562 cells at a 1:5 effector-to-target (E:T) ratio for 72h. (n=3/8) **(B)** Flow cytometric analysis revealing the NKG2D surface expression on activated NK-92MI cells. (n=4) **(C)** The percentage of NKG2D-positive cells following co-culture of MICA-transfected MRC-5 human lung fibroblasts with NK-92MI cells. (n=3) **(D)** The expression of MICB was observed when activated NK-92MI cells were co-cultured with HLF-1 fibroblasts. (n=3) **(E, F)** The expression of fibronectin and TGF-β1 was observed when activated NK-92MI cells were co-cultured with HLF-1 fibroblasts. (n=3) The data are presented as the mean ± SEMs; *P<0.05, **P<0.01, ***P<0.001.

On the basis of these observations, we investigated NK cell–fibroblast interactions via *in vitro* coculture systems. NK-92-MI cells were activated with IL-2 or cocultured with K562 chronic myeloid leukemia cells at a 1:5 effector-to-target ratio for 72 hours. Functional analysis revealed significant NK cell activation, marked by increased IFN-γ secretion, increased LDH release (**Figure 2A**), and a substantial increase in NKG2D^+^ cell populations, as detected via flow cytometry (**Figure 2B**). Collectively, these data confirm that K562 stimulation potently activates NK cells while increasing surface NKG2D expression.

Coculture with MICA-transfected MRC-5 fibroblasts further amplified NKG2D expression on NK cells (**Figure 2C**), demonstrating ligand-induced receptor modulation. Similarly, when activated NK-92MI cells (pre-stimulated with K562) were cocultured with HLF-1 fibroblasts, MICA/B expression increased (p<0.001, **Figure 2D**; **Supplementary Figure 2**). Concurrently, the levels of the fibrotic markers fibronectin and TGF-β were elevated in these coculture systems (**Figures 2E, F**), suggesting that bidirectional NK-fibroblast communication through the NKG2D-MICA/B axis may drive fibrotic progression.

### 3.3 NKG2D-AAV5 induces fibrosis progression in PF model mice

**Figure 3:**
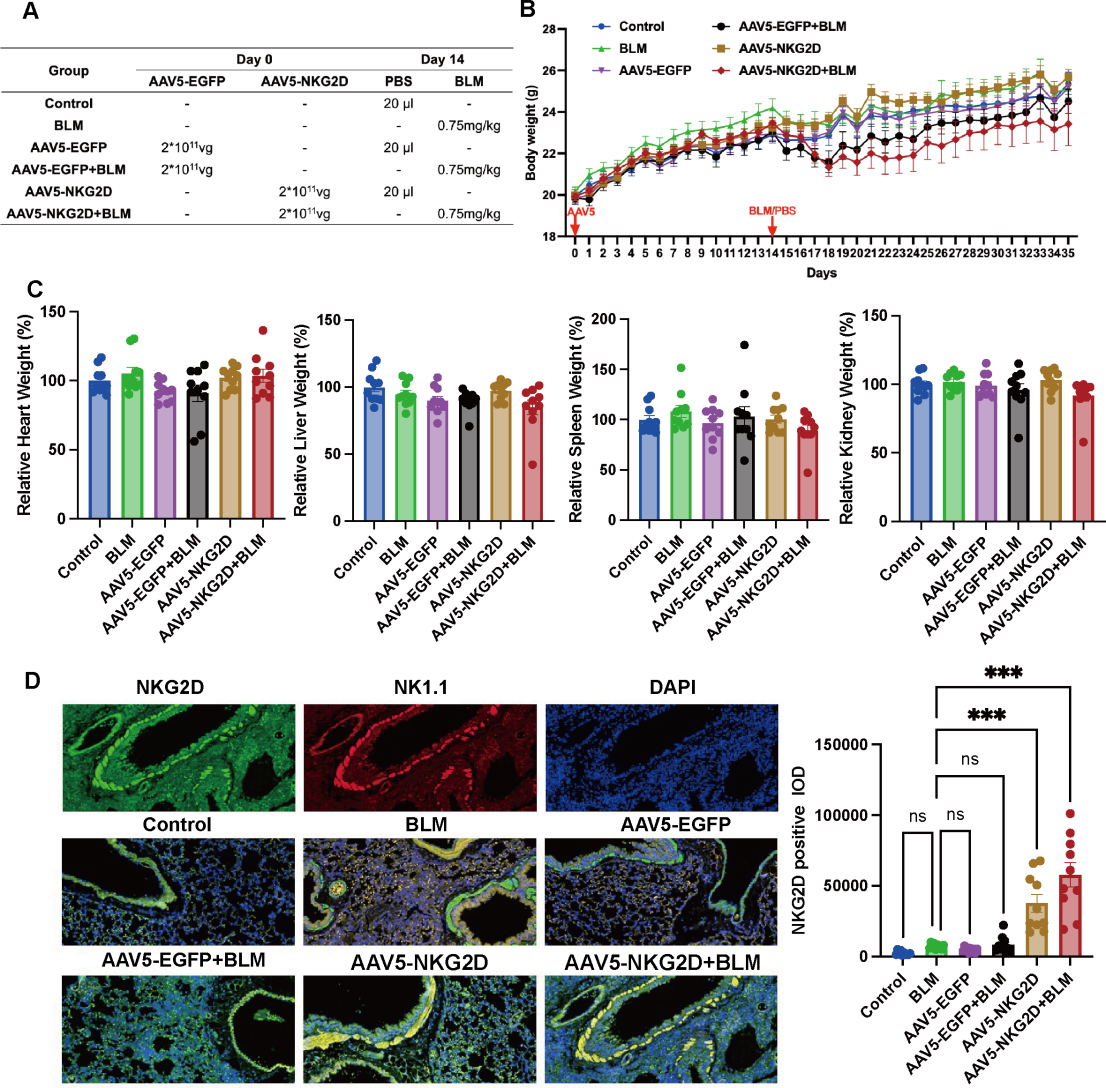
Construction of NKG2D-AAV5 mice. **(A)** The different processing procedures for each group of mice. **(B)** Body weight curve of mice. (n=10) **(C)** Weight diagrams of the heart, liver, spleen, and kidney organs of mice in each group. (n=10) **(D)** IF was used to detect the co-localization of NKG2D and NK1.1 proteins in mouse lung tissue, and semi-quantitative analysis of NKG2D expression was conducted. (n=10) The data are presented as the mean ± SEMs; *P<0.05, **P<0.01, ***P<0.001.

Our innovative animal model combining NKG2D-AAV5 with BLM induction provides compelling evidence for the pathogenic role of NKG2D. Notably, no significant differences in body weight or organ weight were observed among the experimental groups of mice, suggesting a favorable safety profile for NKG2D-AAV5. IF analysis of whole lung sections revealed colocalization of the NKG2D protein (red fluorescence) and NK1.1^+^ cells (green fluorescence), as evidenced by merged yellow signals. This result specifically confirms that NKG2D expression is restricted to NK cells within murine lung tissue (**Figure 3D**). Compared with that in the BLM group, the fluorescence intensity of NKG2D at positive sites in both the NKG2D-AAV5 group and the NKG2D-AAV5+BLM combination group was significantly greater (**Figure 3D**). Collectively, these findings validate the efficient and targeted expression of NKG2D via AAV5 transduction, as well as its precise functional localization to NK cells in this experimental setting.

**Figure 4:**
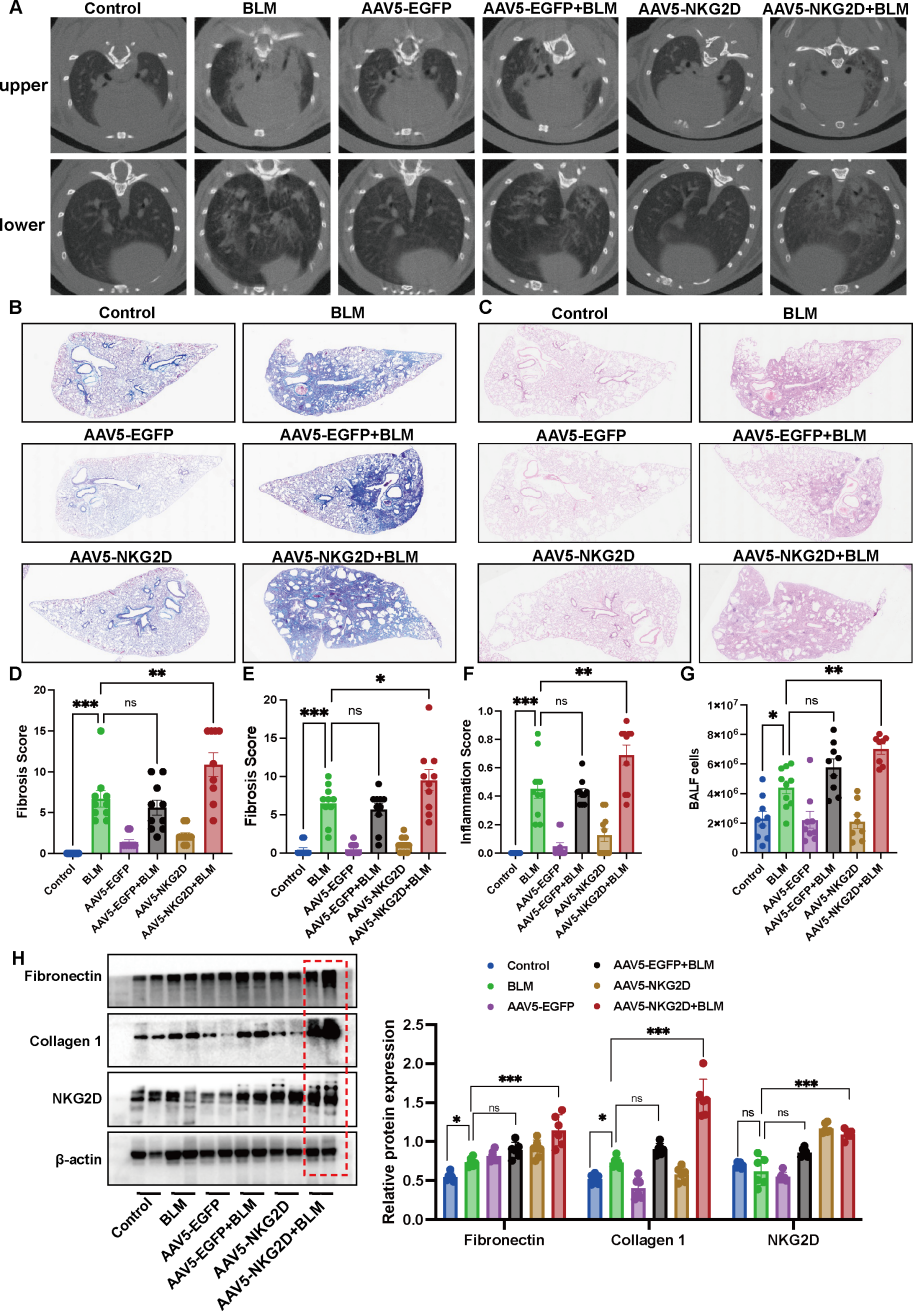
NKG2D-AAV5 induces fibrosis progression in PF mice. **(A, D)** Analysis of CT imaging images and fibrosis scores of mice in each group. (n=10) **(B, E)** Masson staining and semiquantitative analysis of the images of lung tissue in each group mice. (n=10) **(C, F)** HE staining and semiquantitative analysis of the images of lung tissue in each group mice. **(G)** Count of BALF cells in each group of mice. (n=8-10) **(H)** Detection of fibrosis index proteins in lung tissues of mice in each group. (n=6) The data are presented as the mean ± SEMs; *P<0.05, **P<0.01, ***P<0.001.

Advanced imaging and histological analyses provided robust evidence of disease exacerbation. CT scans and Masson’s trichrome staining revealed significantly higher fibrosis scores in the NKG2D-AAV5+BLM group than in the BLM-alone group (**Figures 4A, B, D, E**). Furthermore, compared with the control group, the combined use of AAV5-NKG2D and BLM significantly increased the protein expression of hydroxyproline in mouse lung tissue (**Supplementary Figure 3**). Consistently, H&E staining confirmed elevated inflammatory infiltration in the NKG2D-AAV5+BLM group (**Figures 4C, F**). Molecular analyses further supported these findings, with BALF cell counts significantly increased in the NKG2D-AAV5+BLM group (**Figure 4G**). WB analysis revealed markedly increased protein expression of collagen-I and fibronectin in NKG2D-AAV5+BLM-treated lungs (**Figure 4H**). These results collectively establish that NKG2D activation, delivered efficiently and specifically by AAV5, synergizes with BLM-induced injury to accelerate PF through multiple pathological cascades.

### 3.4 NKG2D overexpression drives fibrosis via the DAP12-SYK axis and induces cellular senescence

**Figure 5:**
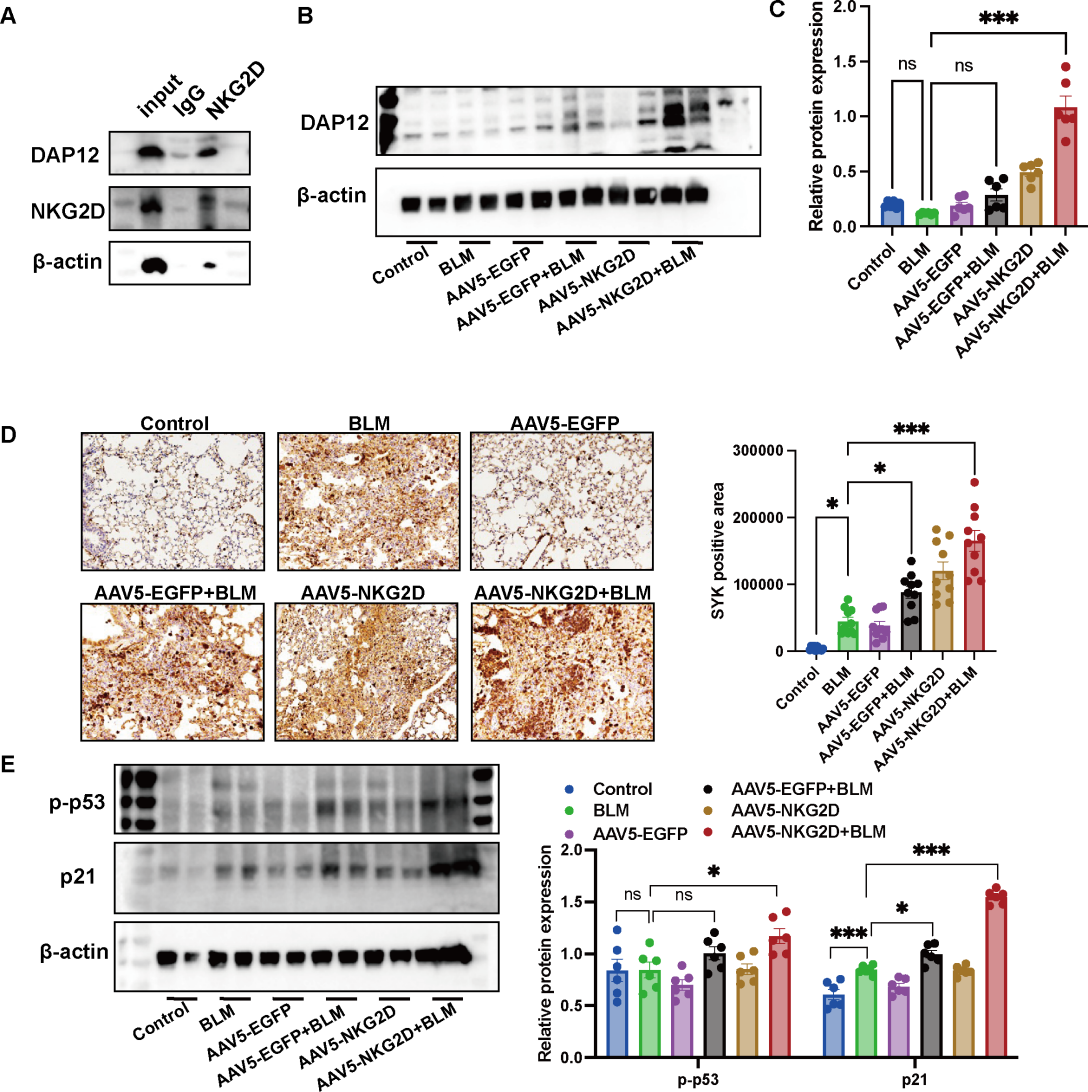
Detection of NKG2D-AAV5 induced cellular aging in mice. **(A)** CO-IP analysis of the interaction between NKG2D and DAP12 proteins. (n=3) **(B, C)** WB analysis and semi-quantitative analysis of DAP12 protein expression in lung tissues of mice in each group. (n=6) **(D)** IHC analysis and semi-quantitative analysis of SYK protein expression in lung tissues of mice in each group. (n=10) **(E)** WB analysis and semi-quantitative analysis of p-p53 and p21 protein expressions in lung tissues of mice in each group. (n=6) The data are presented as the mean ± SEMs; *P<0.05, **P<0.01, ***P<0.001.

Mechanistically, co-immunoprecipitation assays in K562 cells confirmed a robust interaction between NKG2D and its adaptor protein DAP12 (**Figure 5A**). *In vivo*, protein expression of DAP12 was significantly increased in the NKG2D-AAV5+BLM group (**Figures 5B, C**), validating the relevance of this axis. Analysis of downstream signaling revealed pronounced upregulation in the combination group: immunohistochemistry showed elevated SYK expression (**Figure 5D**), while Western blotting detected increased levels of the senescence-associated markers phospho-p53 (Ser15) and p21 (**Figure 5E**). These findings indicate that NKG2D overexpression accelerates PF by activating the DAP12-SYK-p53-p21 cellular senescence axis.

### 3.5 Therapeutic blockade of NKG2D alleviates BLM-induced pulmonary fibrosis

**Figure 6:**
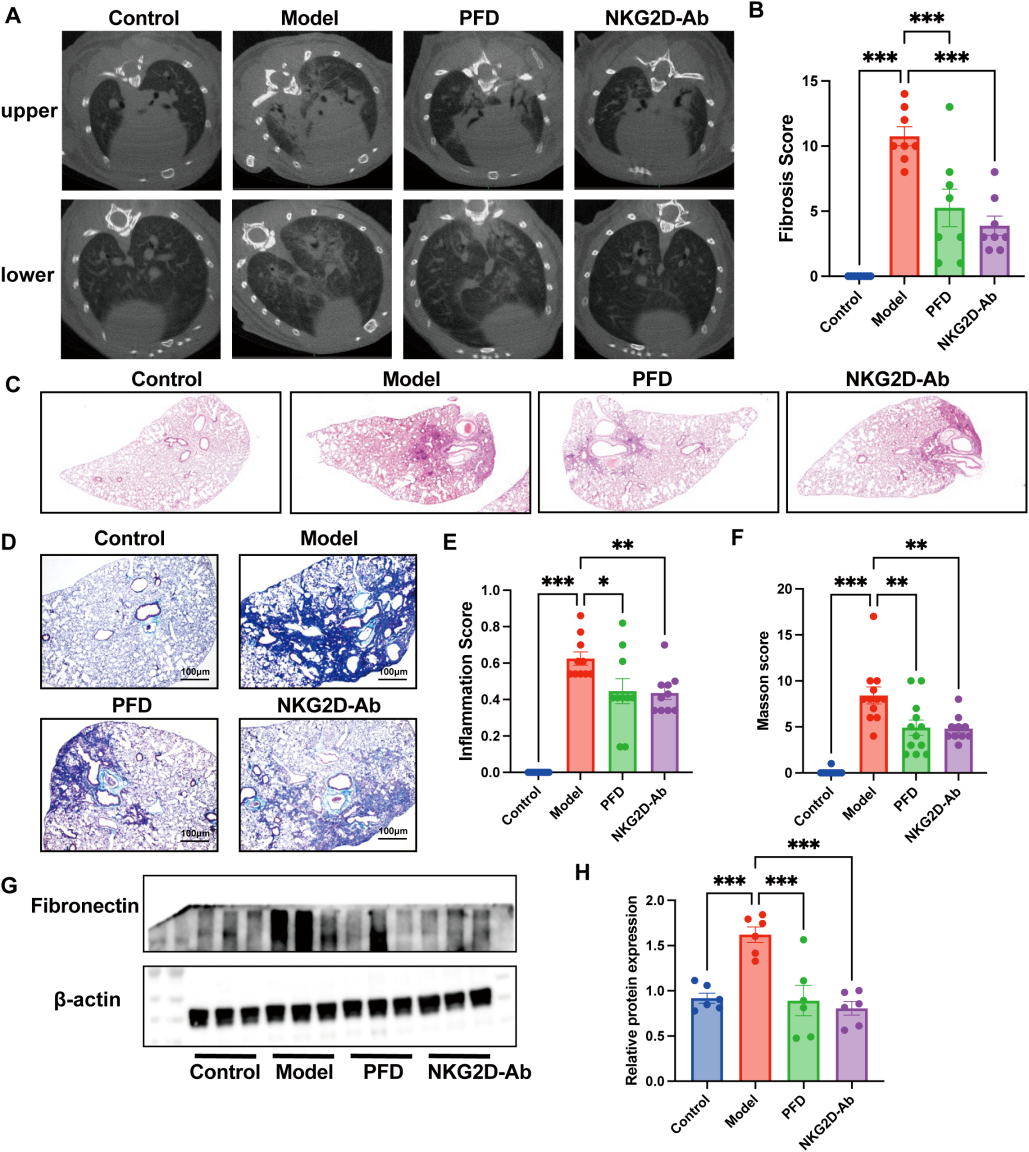
Therapeutic efficacy assessment of anti-NKG2D antibody in BLM-induced PF. **(A, B)** Analysis of CT imaging images and fibrosis scores from NKG2D-Ab treated PF mice. (n=8) **(C, E)** HE staining and semiquantitative analysis of the images of lung tissues from NKG2D-Ab treated PF mice. (n=10) **(D, F)** Masson staining and semiquantitative analysis of the images of lung tissues from NKG2D-Ab treated PF mice. (n=10/12) **(G, H)** WB analysis and semi-quantitative analysis of fibronectin protein expression in lung tissues from NKG2D-Ab treated PF mice. (n=6) The data are presented as the mean ± SEMs; *P<0.05, **P<0.01, ***P<0.001. PFD, pirfenidone.

Therapeutic intervention with an anti-NKG2D monoclonal antibody in BLM-induced PF mice significantly ameliorated disease progression. Micro-CT quantification showed an approximately 40% reduction in fibrotic lesion volume compared to the BLM model group (**Figures 6A, B**); histopathological evaluation indicated attenuated lung inflammation and diminished collagen deposition following antibody treatment (**Figures 6C–F**); and WB further confirmed significant downregulation of the fibrosis marker fibronectin in treated versus untreated PF mice (**Figures 6G, H**). Collectively, these integrated findings demonstrate that anti-NKG2D antibody therapy effectively reduces PF progression through multifaceted modulation of inflammatory and fibrotic pathways.

**Figure 7:**
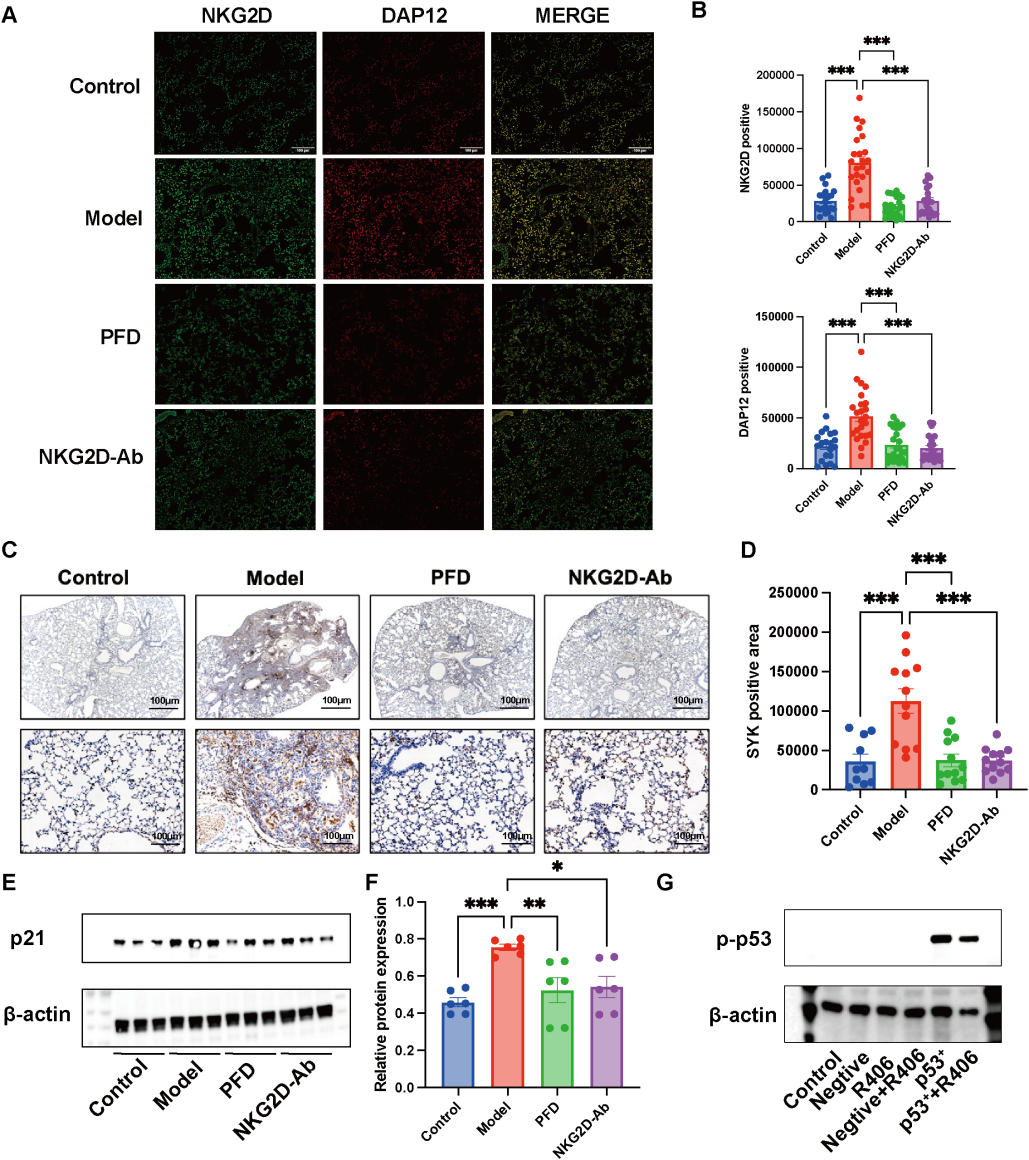
Assessed downstream signaling molecules of NKG2D in lung tissues from NKG2D-Ab treated PF mice. **(A, B)** Localization of co-expression of NKG2D and DAP12 in lung tissues of mice in each group and semi-quantitative analysis of NKG2D and DAP12 protein. (n=20/24) **(C, D)** IHC analysis and semi-quantitative analysis of SYK protein expression in lung tissues of mice in each group. (n=10/12) **(E, F)** WB analysis and semi-quantitative analysis of and p21 protein expressions in lung tissues of mice in each group. (n=6) **(G)** Effect of SYK inhibition on p53 expression. (n=3) The data are presented as the mean ± SEMs; *P<0.05, **P<0.01, ***P<0.001. PFD, pirfenidone.

We further assessed downstream signaling molecules of NKG2D in lung tissues from PF mice before and after therapeutic intervention. IF analysis revealed significant colocalization of NKG2D and DAP12 proteins in fibrotic lungs, with both proteins markedly downregulated (**Figures 7A, B**) following anti-NKG2D antibody treatment. Consistently, IHC and WB demonstrated concomitant suppression of SYK and p21 expression (**Figures 7C–F**), indicating effective blockade of the NKG2D-DAP12-SYK-p53 signaling axis. To investigate the role of SYK in regulating p53, we performed an experiment in HEK293T cells overexpressing p53, treating them with the SYK inhibitor R406. Western blot analysis revealed that R406 treatment significantly reduced p53 protein levels (**Figure 7G**). This result suggests that SYK activity may positively regulate p53 expression.

## 4 Discussion

**Figure 8:**
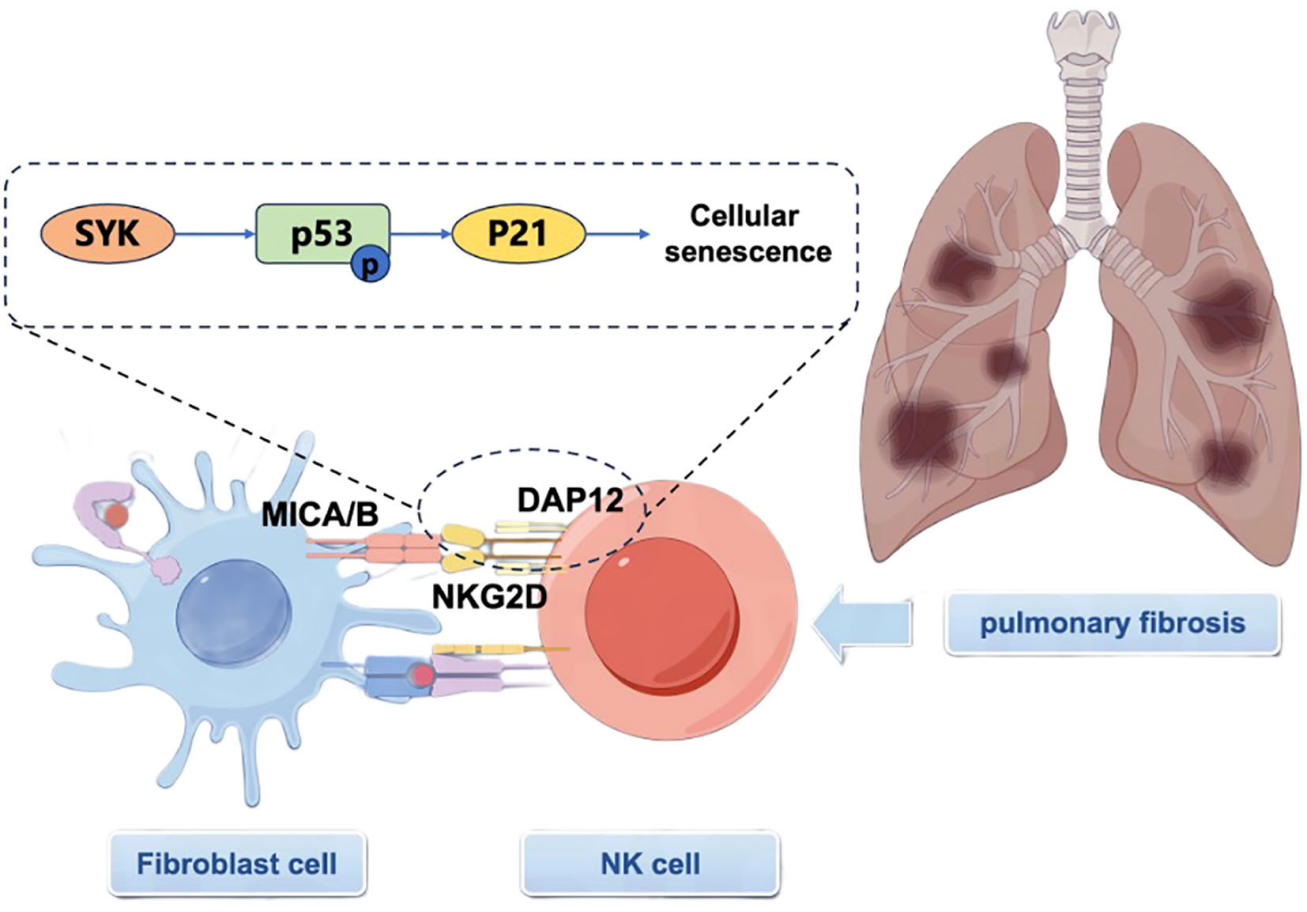
Diagram of the mechanism of action of NKG2D-DAP12-SYK-P-P53-P21 in PF diseases.

Our study elucidates a critical role for the NKG2D-MICA/B axis in the pathogenesis of PF, demonstrating its dual functionality in both immune activation and fibrotic progression. Through BLM-induced model mice, we demonstrated marked upregulation of NKG2D expression in lung NK cells, accompanied by increased levels of NKG2D ligands (MICA/B) on fibroblasts. Mechanistically, we identified the DAP12-SYK-p53-p21 signaling axis as a central mediator of the pro-fibrotic effects of NKG2D. SYK kinase activation induced p53 phosphorylation and p21 upregulation, culminating in senescence, a process mechanistically linked to the SASP. Notably, antibody-mediated blockade of NKG2D attenuated fibrosis, supporting its potential as an immunomodulatory target in PF (**Figure 8**).

The paradoxical effects of NKG2D across different organ systems highlight the critical influence of tissue-specific signaling pathways. Our findings demonstrate that in PF, NKG2D drives fibrogenesis through a DAP12-SYK-dependent mechanism, promoting cellular senescence rather than immune-mediated clearance. This contrasts sharply with its protective role in renal fibrosis, where NKG2D modulates regulatory T-cell (Treg) activity (23, 24), and in liver fibrosis, where it enhances NK cell cytotoxicity against activated hepatic stellate cells (HSCs) (25, 26). These divergent outcomes likely arise from differences in adaptor protein usage (DAP10 vs. DAP12), local immune microenvironments, and ligand diversity (27, 28). The preferential engagement of DAP12 in PF, which leads to SYK activation and subsequent cellular senescence, underscores the need for tissue-specific therapeutic strategies for fibrotic diseases. Future studies should explore conditional knockout models and DAP12 ablation to further validate the necessity of this pathway while also investigating how microenvironmental cues dictate NKG2D functional plasticity across organs. These insights not only refine our understanding of fibrotic pathogenesis but also emphasize the challenges and opportunities in targeting immune–fibrotic crosstalk for therapy.

This study still has several limitations that need to be acknowledged and can provide directions for future research. Notably, the single-dose bleomycin animal model employed herein induces acute, self-limiting lung injury, and the experiments were conducted on young mice. Therefore, this model cannot fully recapitulate the typical chronic progressive course of human pulmonary fibrosis (29), nor can it adequately reflect the critical aging-related microenvironment involved in disease initiation and progression. This limitation may, to some extent, affect the translation and extrapolation of our findings to clinical contexts. For example, the model fails to replicate the persistent progressive fibrosis with its characteristic basilar and subpleural distribution seen in human PF, and does not induce significant alveolar epithelial remodeling (30, 31). Future studies are warranted to further validate the role of this pathway in chronic fibrosis models or aging animal systems to enhance its clinical relevance.

Second, the efficacy evaluation of the anti-NKG2D antibody in this study only used a single-dose regimen and lacked multi-dose gradient experiments. Therefore, the optimal therapeutic window and dose-response relationship of this antibody are not yet clear, making it difficult to comprehensively assess its clinical efficacy potential and safety profile. Future translational research needs to systematically optimize the dosing strategy through multi-dose gradient experiments to clarify the therapeutic window and dose-response characteristics of this antibody, and further explore its potential for combination therapy with existing anti-fibrotic drugs or SYK inhibitors, thereby providing more solid experimental evidence for clinical intervention targeting this pathway in pulmonary fibrosis.

Finally, although we confirmed that the upregulation of the NKG2D/DAP12/SYK pathway specifically targets NK cells, the activation of the p53-p21 pathway was only verified at the whole lung tissue level, and its specific location at the cellular level has not yet been clarified—that is, whether this activation occurs in fibroblasts, NK cells, or other lung cell subsets. This crucial question remains unresolved, limiting in-depth analysis of the regulatory network of this pathway. Future research should utilize cell colocalization techniques and cell-specific gene manipulation models to precisely elucidate the cell type-specific regulatory network of the NKG2D/DAP12/SYK-p53-p21 signaling axis and clarify the core roles of each cell subset within the pathway. Furthermore, the NKG2D pathway function revealed in this study contrasts sharply with its classic protective effects in tumor immune surveillance and liver fibrosis, highlighting the significant context-dependent nature of this pathway’s function and suggesting that its mechanism of action may fundamentally change depending on the lesion type and tissue microenvironment.

This study reveals for the first time the crucial role of the NKG2D-DAP12-SYK-p53 signaling axis in pulmonary fibrosis, providing a new perspective for understanding the immune-fibrotic interaction. Future work should further validate the sustained activation mechanism of this pathway in chronic fibrosis or aged animal models and clarify its cellular localization using cell-specific manipulation techniques. Simultaneously, optimizing NKG2D targeted intervention strategies (such as dose windows and combination therapies) will promote the clinical translation of this pathway and provide new immunomodulatory approaches for the treatment of pulmonary fibrosis.

## 5 Conclusion

The NKG2D-MICA/B axis contributes to pulmonary fibrosis in the bleomycin-induced model potentially through a DAP12-SYK-p53-p21-mediated cellular senescence pathway. While NKG2D overexpression exacerbated fibrotic responses, its blockade significantly attenuated pathology. The therapeutic effect of NKG2D blockade appeared distinct from and, in this model, more pronounced than that of pirfenidone, suggesting a dual mechanism targeting both immune activation and cellular senescence. Overall, these preclinical research results suggest that the NKG2D pathway may be a novel immune fibrosis regulatory factor.

## Data Availability

The original contributions presented in the study are included in the article/**Supplementary Material**. Further inquiries can be directed to the corresponding authors.
