# Supplementary material for: Interaction between NKG2D and its ligands MICA/B activates the DAP12/SYK/p53/p21 axis to drive pulmonary fibrosis
Source: Front Immunol. 2026 Mar 2;17:1770733. doi: 10.3389/fimmu.2026.1770733 (PMC12989593; doi:10.3389/fimmu.2026.1770733)
Supplement: Supplementary file 1 [file Table1.docx]

**Supplementary Files**

Preliminary results from experiments conducted before the formal experiments showed that when adeno-associated virus type 5 (AAV5) injection was combined with a standard dose of bleomycin (BLM, 1.5 mg/kg), mice exhibited extremely high mortality rates (specific percentages or fractions, >70% within 15 days). These results suggested that this combination regimen had unacceptable toxicity in animals. Therefore, in the subsequent formal 35-day AAV5 synergistic model, we adjusted the BLM dose to 0.75 mg/kg, thereby significantly reducing animal mortality while maintaining model effectiveness, enabling the completion of the long-term experiment.


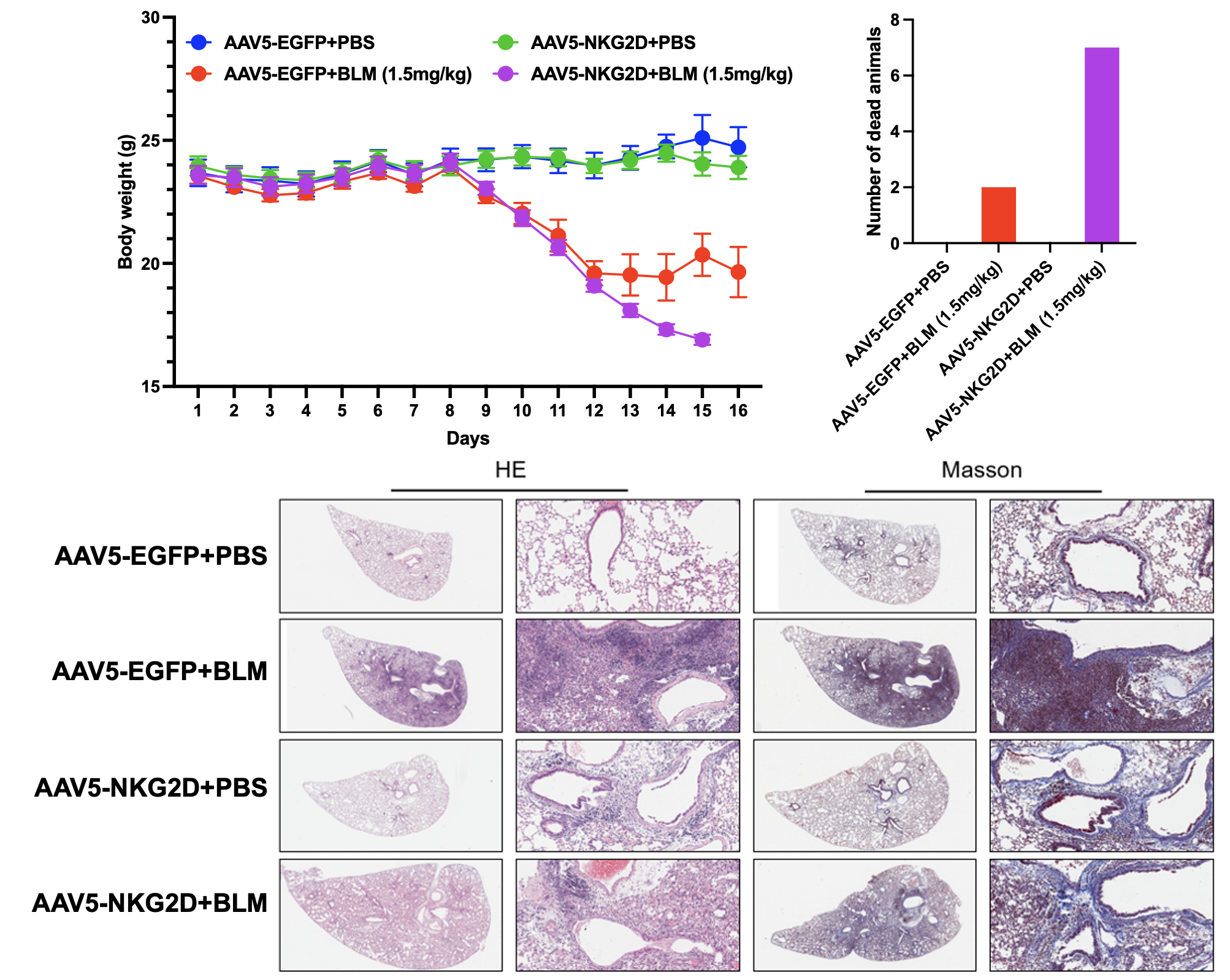


SFig 1. Preliminary experiment: Combined use of AAV5 with standard dose of BLM (1.5 mg/kg) led to high mortality.


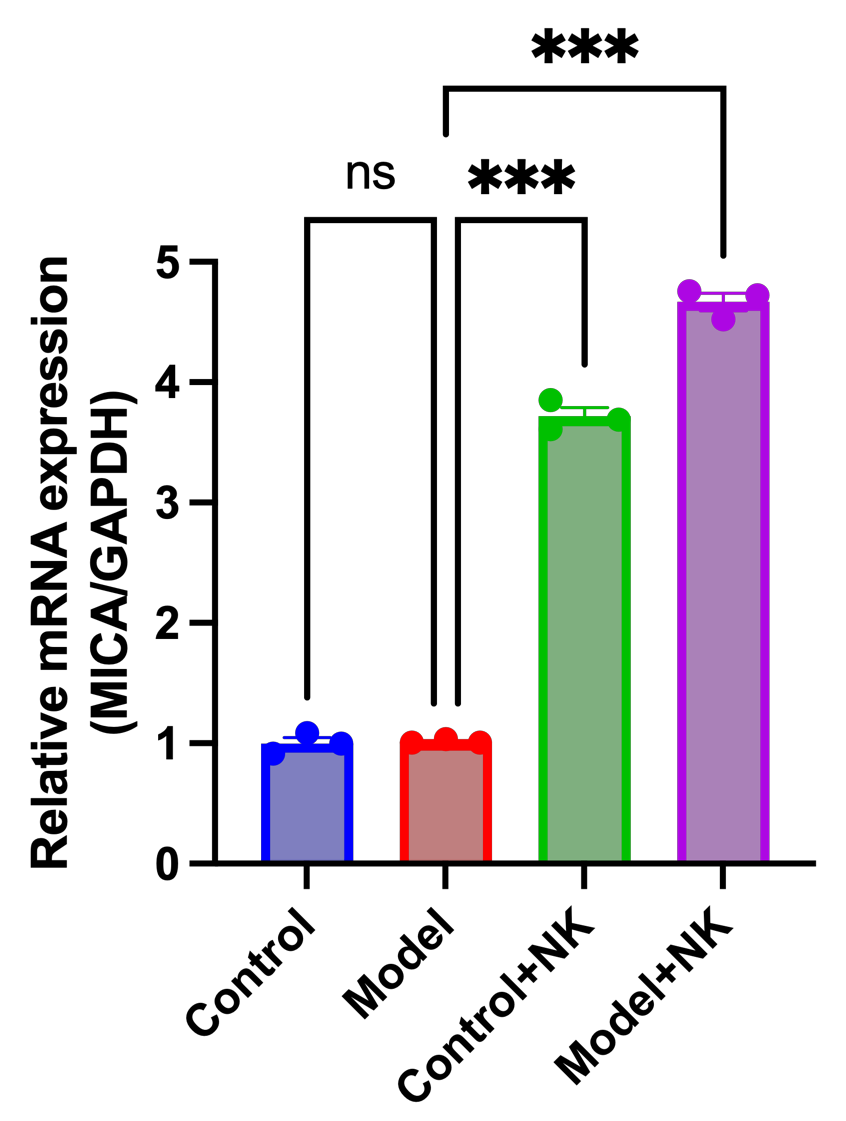


SFig 2. The expression of MICA was observed when activated NK-92MI cells were co-cultured with HLF-1 fibroblasts.

SFig 3. HYP expression in lung tissue of AAV5-induced mouse model.
