# Supplementary material for: Interaction between NKG2D and its ligands MICA/B activates the DAP12/SYK/p53/p21 axis to drive pulmonary fibrosis
Source: Front Immunol. 2026 Mar 2;17:1770733. doi: 10.3389/fimmu.2026.1770733 (PMC12989593; doi:10.3389/fimmu.2026.1770733)

fig.1A

fibronectin β-actin


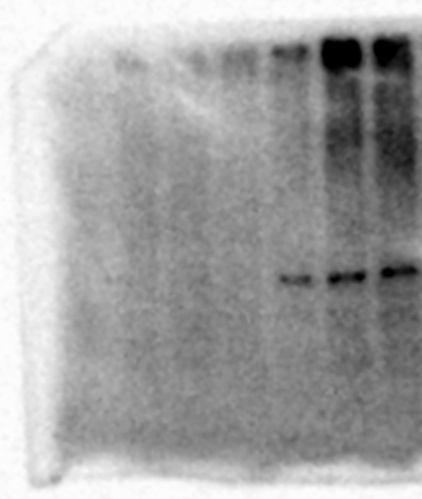

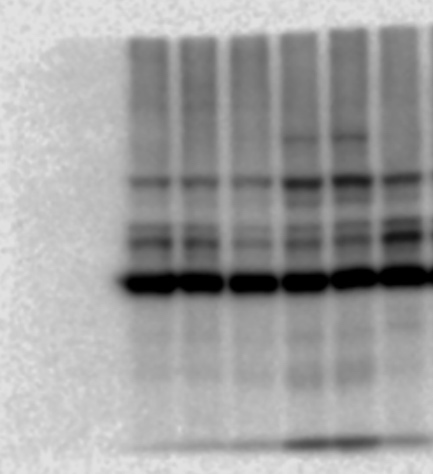


nkg2d β -actin


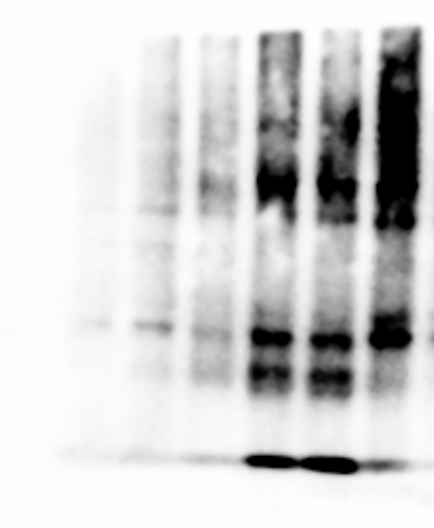

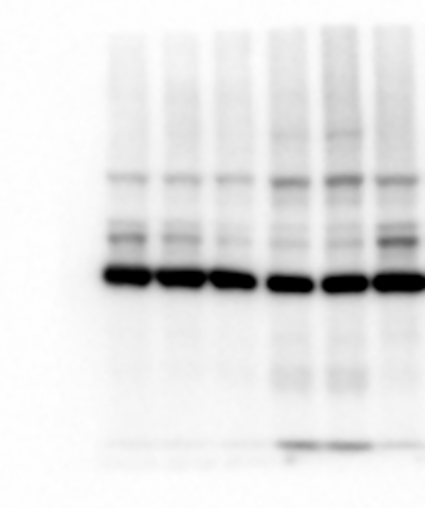


fig.2E

fibronectin GAPDH


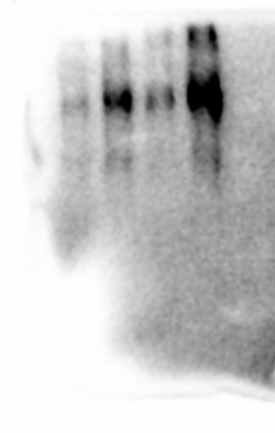

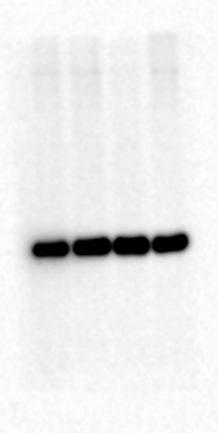


fig.4H

fibronectin collagen-1


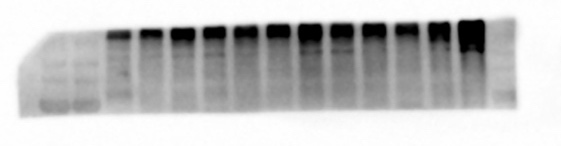

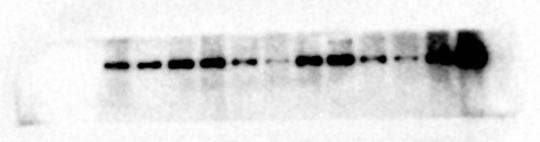


nkg2d β-actin


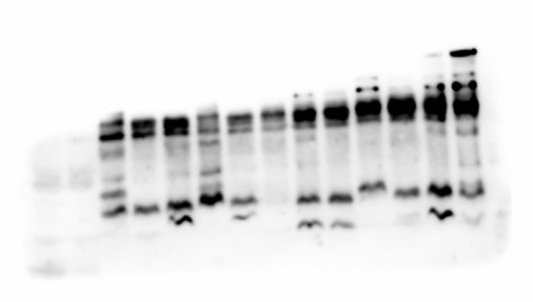

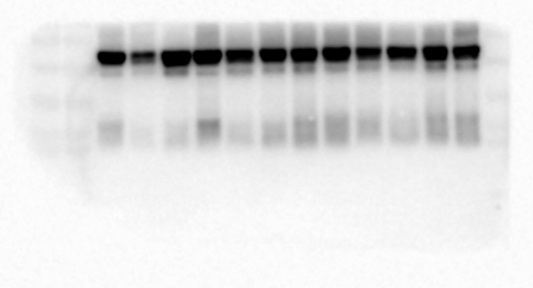


all


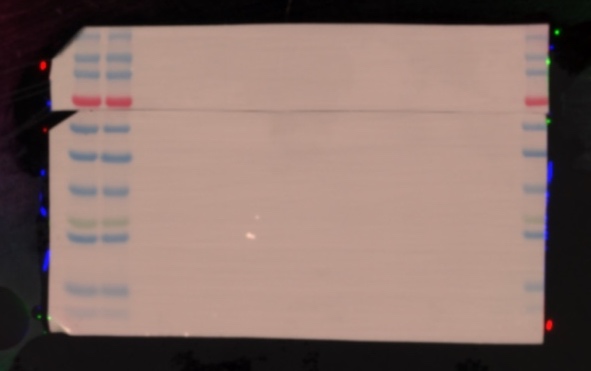


fig.5B

dap12 β -actin


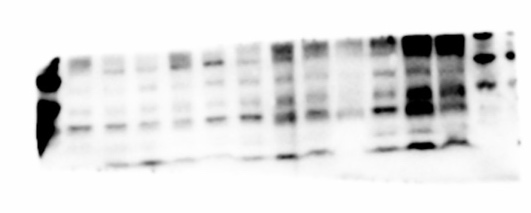

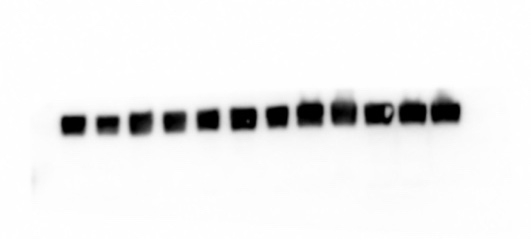


all


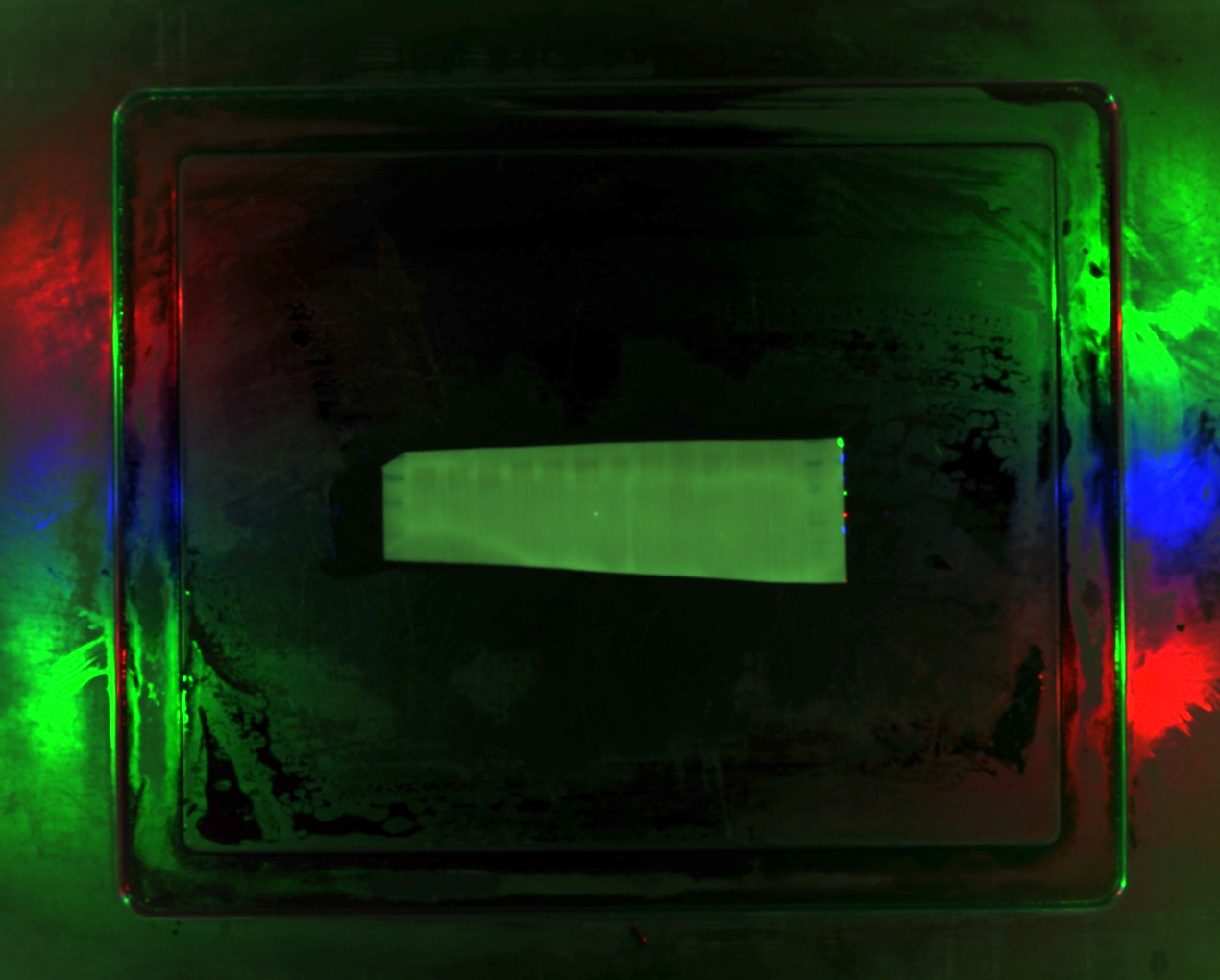


fig.5E

p-p53 p21


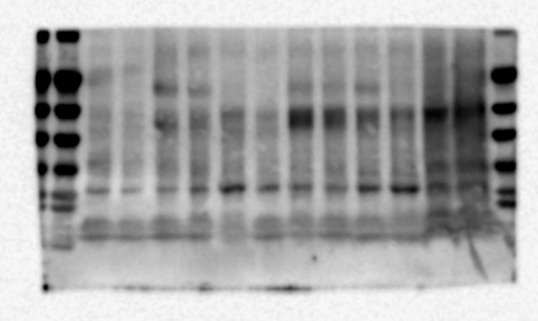

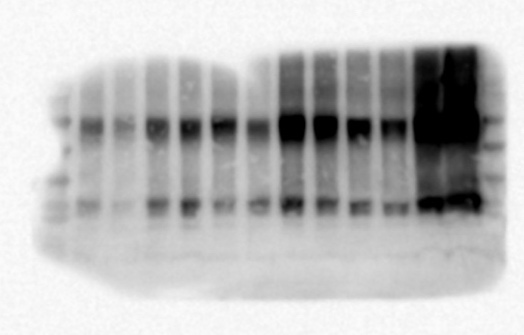


β -actin


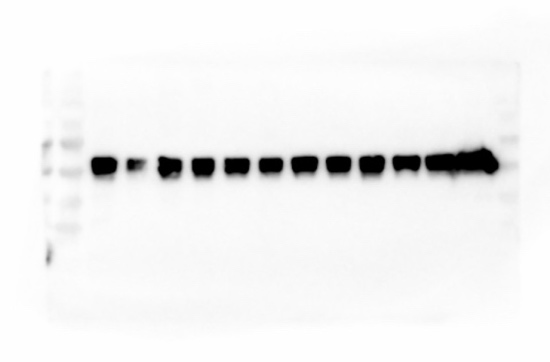


fig.6G

fibronectin β -actin


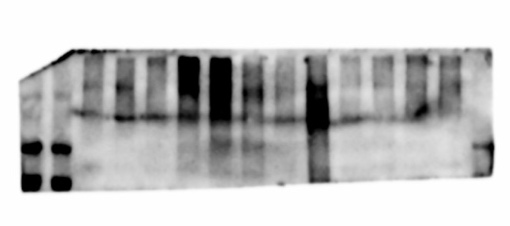

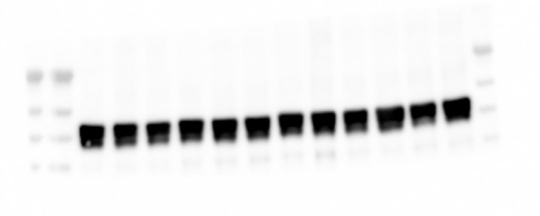


all


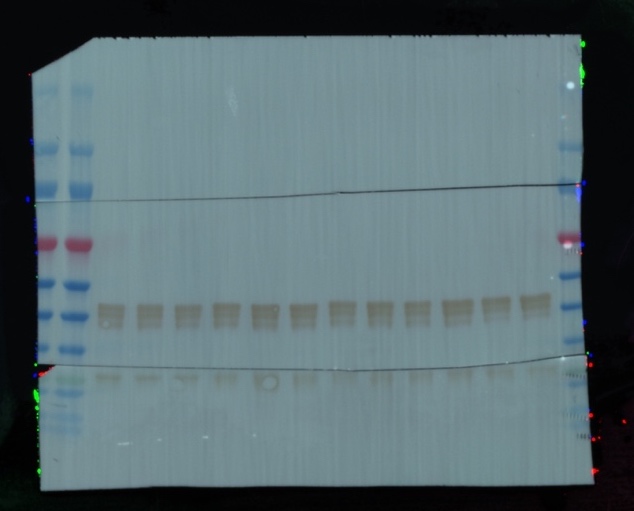


fig.7E

dap12 b-actin


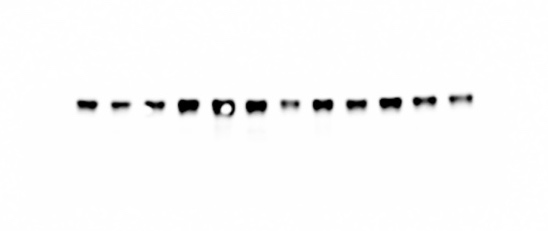

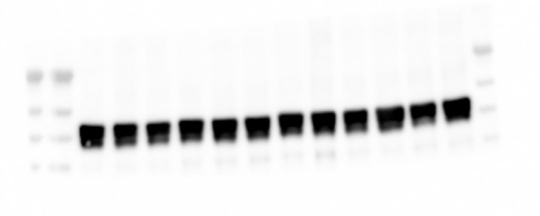


all


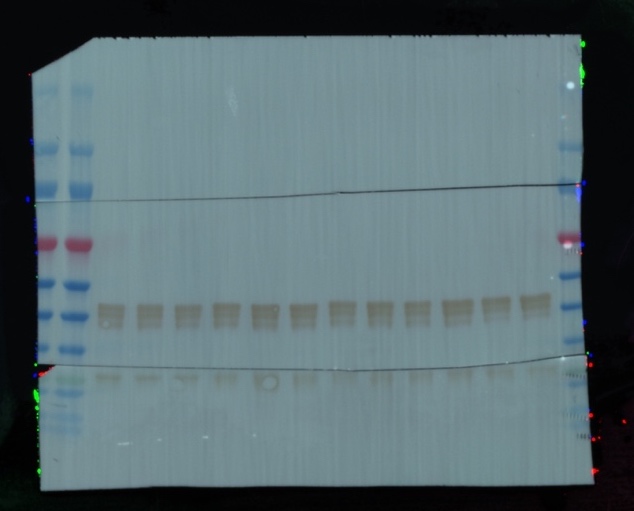

Supplement: Supplementary file 2 [file DataSheet1.docx]
